# Students’ perceptions of and satisfaction with their Orthopaedic posting learning environment by using the Healthcare Education Micro-Learning Environment Measure (HEMLEM) questionnaire

**DOI:** 10.1371/journal.pone.0306971

**Published:** 2024-07-11

**Authors:** Syeda Rubaba Azim, Syed Muhammad Azfar, Mukhtiar Baig

**Affiliations:** 1 Department of Medical Education, Dow University of Health Sciences, Karachi, Pakistan; 2 Department of Orthopedic, Liaquat College of Medicine and Dentistry, Karachi, Pakistan; 3 Department of Clinical Biochemistry, Faculty of Medicine, Rabigh, King Abdul Aziz University, Jeddaah, Saudi Arabia; Maragheh University of Medical Sciences, ISLAMIC REPUBLIC OF IRAN

## Abstract

**Background:**

The learning environment in medical education is crucial for student development, encompassing social, psychological, and physical aspects that significantly affect learning. This study aimed to assess undergraduate medical students’ perception of the orthopaedic ward’s learning environment and examine the factors influencing their overall satisfaction during clinical rotation.

**Methods:**

This cross sectional quantitative study was conducted in a private medical college in Pakistan. Data was collected through a pre-validated questionnaire, "The Healthcare Education Micro-Learning Environment Measure (HEMLEM)." Data analysis was done using SPSS version 23 software.

**Results:**

A total of 205/300 students (response rate 68.33%) [103 (50.2%) males and 102(49.85) females] participated in this survey. Notably, 116 (56.6%) appreciated the ward’s welcoming, friendly, and open atmosphere, and 114(55.6%) of the respondents appreciated the ward culture where they felt free to ask questions or comment. Additionally, 111(54.7%) appreciated the faculty’s enthusiasm for teaching. A comparison between male and female students showed significantly higher satisfaction among males regarding staff attitudes and behaviours (p < .019).

**Conclusion:**

Undergraduate students held a predominantly positive view of the orthopaedic ward’s learning environment, with differences observed based on gender and year of study. The study highlights the importance of both staff attitude and teaching quality in shaping the educational experience. It suggests that medical institutions should focus on enhancing teaching skills among clinicians to improve learning experiences and ultimately benefit patient care and the healthcare system.

## Introduction

The educational environment is the social, psychological, and physical context in which a learner learns [1]. The significance of the learning environment of an educational institute has gained much attention in terms of its effect on learning [2]. A supportive learning environment improves students’ learning experience, promoting student empathy, professionalism, and academic rigor and enhancing the student’s well-being. In contrast, an unaccommodating learning environment may lead to increased mental distress, decreased learning, exhaustion, and burnout [3, 4].

Clinical rotations are increasingly valued as an important setting for learning within health professions education [5]. In clinical rotations, learners engage with real-life problems that enhance their motivation and engagement in learning. Teachers have a crucial role in modelling professional attitudes and behaviour, facilitating the integration of essential skills such as history-taking, physical examination, clinical reasoning, decision making, empathy, and professionalism [6]. The clinical learning environment represents a multifaceted entity shaped by individual, interpersonal, and organizational elements. It forms a mosaic of diverse and continuously changing microenvironments that learners experience throughout their training [7].

Medical institutions frequently presuppose that medical students achieve necessary learning outcomes merely through participation in clinical postings. Undergraduate students commence their clinical education encountering scenarios in ward settings, where teaching tends to be spontaneous, ad hoc, and context-specific [8]. In a clinical setting, two important factors may influence students’ learning [9]. The first one is the attitude and behaviour of teachers and the support provided by their peers in the workplace, which is critical for students’ competence development and psychological well-being [10]. The second factor is teaching quality, as clinical teachers are responsible for providing a safe and constructive learning environment by incorporating role-modelling, scaffolded guidance, and constructive feedback in their teaching practices [11, 12]. Both elements play a significant role in learners gaining knowledge through diverse teaching methods. Nevertheless, the attitude of the staff, effective role modelling, and a nurturing environment are equally essential for enhancing student learning in a ward setting [13].

The Orthopaedic rotation is one of the most important clinical postings for undergraduate medical students. It is observed that undergraduate medical students often do not take much interest in this specialty compared to other main clinical subjects in the undergraduate medical curriculum, such as surgery and medicine [14]. The Orthopaedic ward offers a significant educational opportunity for undergraduate medical students to acquire and improve crucial skills [15]. However, due to the simultaneous provision of service and teaching, it seems difficult to create sufficient learning opportunities [16].

While the significance of the clinical learning environment is well recognized, much of this research has mainly focused on nursing or postgraduate medical students [4, 17, 18]. A study by Sellburg (2021) has examined the clinical learning environment in various subjects among undergraduate medical professionals. It has primarily assessed the broader institutional learning environment, neglecting crucial micro-level factors such as staff attitudes and teaching quality [5]. Additionally, there remains a lack of detailed understanding regarding how undergraduate medical students perceive the micro learning environment particularly in orthopaedics wards. This study was planned to evaluate undergraduate medical students’ perception of the orthopaedic ward’s learning environment and explore the factors in the clinical learning environment that contributed to the student’s satisfaction during their clinical rotation. Based on the results of this survey, various modifications in staff attitudes and behaviours and teaching quality may be advised to improve the educational environment during clinical rotations, with the intention that students will better serve the community.

The significance of the clinical learning environment for undergraduate medical students, in orthopaedics, has been understudied despite its importance in knowledge transfer and skill development. While existing research has primarily focused on nursing or postgraduate medical students, there is a notable gap in understanding the microlearning environment’s impact on undergraduates, especially in specialized areas like orthopaedics.

To address this gap, our study aimed to evaluate undergraduate medical students’ perceptions of the orthopaedic ward’s learning environment, emphasizing staff attitudes, behaviours, and teaching quality. By focusing on a private medical college in Pakistan, we aimed to offer specific insights into the orthopaedic learning environment.

Our research fills a significant void in the literature by addressing the specific needs and challenges of undergraduate medical students in orthopaedics. By identifying areas for improvement in staff attitudes and teaching quality, we aim to enhance the overall educational experience during clinical rotations, ultimately better practicing professionals to serve their communities.

## Methods

This was a cross-sectional study in which quantitative methodology was adopted. This study was done on Liaquat Medical and Dental College (LCMD) undergraduate medical students in Karachi, Pakistan, between 1st Jan 2021 and April 2021. After receiving ethical approval from the LCMD ethical committee (DSH/IRB/2019/0011), data was gathered through an online survey that included demographic information, such as the year of study and gender. Attached to the questionnaire was a consent form providing essential details about the study. Participation was voluntary, and prospective participants had the freedom to decline or retract their consent at any point.

The study encompassed fourth and fifth-year students selected after completing their Orthopaedics rotation. It included 100 fourth-year students, 100 fifth-year students, and another 100 fifth-year students who had completed their classes and were awaiting exams. Data collection was carried out using the Healthcare Education Micro-Learning Environment Measure (HEMLEM) questionnaire, which was developed by Isba et al. [9]. This questionnaire specifically targeted microlearning environments, such as clinical postings for medical students. Unlike other tools available in the literature that focus on generalized learning environments, the HEMLEM questionnaire is designed to assess small, dynamic environments encountered during clinical placements. This tool is concise and tailored to assess small, dynamic microlearning environments encountered by learners during clinical placements [9].

This tool comprises 12 items divided into two categories: staff attitudes and behaviours and teaching quality. The initial six items focus on staff attitudes and behaviours, aiming to gauge students’ perceptions of these aspects, including the staff’s friendliness, welcoming nature, appreciation of student input, supportiveness, and commitment to students and teaching. The latter six items explore teaching quality, assessing students’ views on aspects such as how much teaching enhances knowledge and skills development, centres on patient care, and aligns with students’ requirements.

Frequencies and percentages were calculated for categorical variables, whereas means and standard deviations were calculated for continuous variables. According to training years and gender distribution, a comparison of HEMLEM scores was explored using an independent t-test. A value of 0.05 or less was considered statistically significant. All the statistical analyses were performed using SPSS version 23.

## Results

A total of 205/300 students (response rate 68.33%) [103 (50.2%) males and 102(49.85) females] participated in this survey. More than half of the students [118(57.6%)] were from the fifth year, and 87(42.4%) were from the fourth year (Table 1).

**Table 1:** General characteristics of the participants.

Variables	Frequency	Percentage
Gender		
Male	103	50.2
Female	102	49.8
Year of study		
Fourth year	87	42.4
Fifth-year	118	57.6

Statement regarding the placement in the orthopaedic ward as a welcoming, friendly, and open atmosphere was much appreciated by the males than females (p < .001). More than one-third of the students, 116(56.6%), appreciated the ward’s welcoming, friendly, and open atmosphere, and 114(55.6%) of the respondents appreciated the ward culture, where they felt free to ask questions or comment. More than half of the participants, 111(54.7%), appreciated that the faculty was enthusiastic about teaching.

Overall, students’ responses regarding the learning environment in the Orthopaedic ward were positive (Table 2). Almost half of the participants, 99(48.3%), said their knowledge and skills developed in this placement. Similarly, 114(55.6%) of the students admitted that they had the opportunity to apply their previous knowledge in this placement. However, in response to the statements, "Faculty showed an interest in students’ learning." "I was able to meet my learning objectives on this placement," "I had the opportunity to deal with the patient as a whole on this placement," and "I was given tasks suitable for my stage of training on this placement" more than 50% students responded neutral, disagree and strongly disagree (Table 2).

**Table 2:** Students’ evaluation of learning environment of Orthopedic ward.

Statement	SDA 1	DA 2	N 3	A 4	SA 5
This placement had a welcoming, friendly, and open atmosphere.	0	6(2.9)	83(40.5)	80(39)	36(17.6)
There was a culture where I felt free to ask questions or make comments on this placement	3(1.5)	2(1)	86(42)	79(38.5)	35(17.1)
Faculty on this placement were enthusiastic about teaching.	3(1.5)	9(4.4)	82(40)	84(41)	27(13.2)
Faculty showed an interest in students’ learning.	6(2.9)	10(4.9)	97(47.3)	64(31.2)	28(13.7)
Students’ input was valued in this placement	4(2)	11(5.4)	86(42)	74(36.1)	30(14.6)
I was provided with regular, useful, and supportive feedback during this placement.	4(2)	9(4.4)	80(39)	73(35.6)	39(19)
I had the opportunity to apply my previous knowledge in this placement.	7(3.5)	11(5.4)	73(35.6)	82(40)	32(15.6)
My knowledge and skills were developed during this placement.	3(1.5)	13(6.3)	90(43.9)	77(37.6)	22(10.7)
This placement helped me put theory into practice.	5(2.4)	24(11.7)	89(43.3)	56(27.3)	31(15.1)
I was able to meet my learning objectives on this placement.	5(2.4)	19(9.3)	94(45.9)	63(30.7)	24(11.7)
I had the opportunity to deal with the patient as a whole on this placement	9(4.4)	36(17.6)	87(42.4)	49(23.9)	24(11.7)
I was given tasks suitable for my stage of training on this placement.	8(3.9)	34(16.6)	84(41)	57(27.8)	22(10.7)

Strongly disagree(SDA) = 1, Disagree(DA) = 2, Neutral(N) = 3, Agree(A) = 4, Strongly agree(SA) = 5

The male students were substantially happier with the staff attitudes and behaviours than females (p < .019) (Table 3).

**Table 3:** Comparison according to gender and year of study regarding staff attitudes and behaviours and teaching quality.

Variables	Staff attitudes and behaviours Mean ±SD	Teaching quality Mean ±SD
Gender		
Male (n = 103)	22.29±4.23	20.76±5.02
Female (n = 102)	21.09±2.89	20.04±3.56
**P-value**	.019*	.239
Year of study		
Fourth year (n = 87)	22.20±5.14	20.87±5.52
Fifth year (n = 118)	21.32±1.91	20.05±3.23
**P-value**	.135	.217

The fourth-year students much appreciated their placement in the Orthopedic ward, and they admitted that it had a welcoming, friendly, and open atmosphere that was much appreciated than fifth-year students (p < .001) (Table 4). Fourth-year students also responded that there was a culture where I felt free to ask questions or comment on this placement compared to fifth-year students (p = .001) (Table 4).

**Table 4:** Gender-wise and year of study-wise comparison of students’ responses.

	Gender	N	Mean ±SD	Year of study	N	Mean ±SD
This placement had a welcoming, friendly, and open atmosphere.	Male	103	3.90±.834	Fourth year	87	3.93±.938
	female	102	3.52±.685	Fifth year	118	3.55±.608
	P-values	<0.001		P-value	<0.001	
There was a culture where I felt free to ask questions or make comments on this placement	Male	103	3.77±.941	Fourth year	87	3.90±1.023
	female	102	3.61±.662	Fifth year	118	3.53±.580
	P-values	0.163		P-value	0.001	
Faculty on this placement were enthusiastic about teaching.	Male	103	3.71±.925	Fourth year	87	3.64±1.056
	female	102	3.49±.700	Fifth year	118	3.57±.606
	P-values	0.058		P-value	0.393	
Faculty showed an interest in students’ learning.	Male	103	3.50±1.065	Fourth year	87	3.54±1.189
	female	102	3.45±.684	Fifth year	118	3.43±.592
		0.667		P-value	0.516	
Students’ input was valued in this placement	Male	103	3.64±.938	Fourth year	87	3.52±1.109
	female	102	3.48±.805	Fifth year	118	3.59±.657
	P-values	0.190		P-value	0.540	
I was provided with regular, useful, and supportive feedback during this placement.	Male	103	3.77±.982	Fourth year	87	3.67±1.128
	female	102	3.54±.804	Fifth year	118	3.64±.698
	P-values	0.070		P-value	0.859	
I had the opportunity to apply my previous knowledge in this placement.	Male	103	3.69±.970	Fourth year	87	3.66±1.010
	female	102	3.58±.750	Fifth year	118	3.62±.750
	P-values	0.361		P-value	0.766	
My knowledge and skills were developed during this placement.	Male	103	3.53±.937	Fourth year	87	3.53±.998
	female	102	3.46±.699	Fifth year	118	3.47±.676
	P-values	0.527		P-value	0.643	
This placement helped me put theory into practice.	Male	103	3.53±1.018	Fourth year	87	3.49±1.130
	female	102	3.28±.894	Fifth year	118	3.35±.820
		0.064		P-value	0.282	
I was able to meet my learning objectives on this placement.	Male	103	3.46±1.036	Fourth year	87	3.54±1.076
	female	102	3.34±.738	Fifth year	118	3.30±.732
	P-values	0.369		P-value		0.055
I had the opportunity to deal with the patient as a whole on this placement	Male	103	3.28±1.079	Fourth year	87	3.26±1.196
	female	102	3.14±.934	Fifth year	118	3.17±.850
	P-values	0.307		P-value	0.507	
I was given tasks suitable for my stage of training on this placement.	Male	103	3.26±1.093	Fourth year	87	3.39±1.155
	female	102	3.24±.869	Fifth year	118	3.14±.830
	P-values	0.846		P-value	0.076	

Because the questionnaire comprised two components, Cronbach alpha was used to calculate the reliability of both portions. The overall Cronbach’s alpha for the tool was 0.844, with a value of 0.813 for the staff attitudes and behaviours component and 0.875 for the teaching quality aspect.

## Discussion

This study examined the learning environment in the Orthopaedic ward, where students generally valued the ward’s hospitable atmosphere. In a similar vein, Zafar et al.’s study also uncovered a heightened level of contentment among students with the microlearning environments in their four key clinical rotations [4]. It has been noted that students tend to have more positive overall perceptions of the educational environment during their internship phase. This optimistic outlook is attributed to the strengths of learning in a clinical setting centred around real-life clinical scenarios within a professional practice context. The relevance of these experiences to their future profession serves as a motivating factor for the students [8]. Additionally, this setting is distinctive in that it provides a space where both facilitators and students can exemplify professional attitudes and collaboratively engage in learning activities such as history taking, physical examinations, decision-making, clinical reasoning, teamwork, professionalism, and empathy, all integrated seamlessly [6]. Another aspect that enhances the value of the learning environment in the orthopaedic ward is its engaging nature and the provision of new practical skills specific to this field, such as interpreting X-rays and managing fractures, alongside handling real-life scenarios and trauma cases. Adult learners tend to excel when the learning material is made relevant to them. They are particularly keen on subjects that offer immediate benefits in their lives, particularly in areas that affect their societal roles [19].

The study revealed that male undergraduate students exhibited a more favourable reaction to the learning environment of the orthopaedic ward compared to their female counterparts. This could be attributed to a higher interest in orthopaedics among male students. Supporting this observation, a recent report by the Association of American Medical Colleges (AMA) indicated that orthopaedics is the most preferred speciality among male medical residents. In contrast, gynaecology tends to be the speciality most favoured by female residents [20]. Another noteworthy result from this study was the higher level of appreciation expressed by fourth-year students for their orthopedic ward rotation compared to those in their final year. These students acknowledged the placement’s friendly, welcoming, and open environment. This trend suggests that junior learners often exhibit greater satisfaction than their senior peers, possibly due to having lower expectations from their educational experiences [21].

While students demonstrated a positive perception of their learning experience in both sections of the questionnaire, the response to "staff attitude" was notably more favourable than "teaching quality." Research indicates that supportive teacher behaviour greatly influences student motivation. Nonverbal cues from instructors, like smiling, maintaining a positive demeanour, and displaying encouraging facial expressions, significantly enhance the student learning experience [22]. Establishing positive and supportive interactions with learners is recognized as a fundamental aspect of effective teaching [23, 24]. A constructive, nurturing learning environment is an essential part of learning. Staff behaviour and attitude make a student feel comfortable, a place where healthy relationships with peers and teachers flourish [25]. The learning process can be improved by constructing a positive and encouraging atmosphere [26].

Despite an overall positive view of the orthopaedic ward’s learning environment, over half of the students responded neutrally or negatively (disagree or strongly disagree) to statements 4, 10, 11, and 12. Particularly for statement 4, which pertains to the faculty’s interest in student learning, a majority of participants felt that the faculty lacked this interest. This suggests that students who perceive a lack of interest from the teaching staff tend to experience reduced motivation [27]. Conversely, faculty members who encourage students to express their opinions and show a sincere interest in their academic pursuits tend to observe more robust outcomes in student performance [28].

This study found that for three of the four statements, namely 10, 11, and 12 ‐ concerning meeting learning objectives, dealing with patients holistically, and receiving tasks appropriate for their training level ‐ more than half of the responses were neutral, disagree, or strongly disagree. These statements fall under the "teaching quality" category, indicating a need for improvement in this area to better the learning environment in the orthopedic ward. In clinical settings, teachers often juggle multiple roles simultaneously and must adapt to different responsibilities. While most clinical instructors undergo extensive clinical training to develop their knowledge and skills, they frequently receive minimal training in teaching methods [29]. Moreover, Yoo et al. [30] found a significant correlation between learners’ happiness and the learning environment of a medical institute, which ultimately enhances the student’s development as a person as well as a professional. Hence, it falls upon the institution to equip clinicians with vital teaching skills, ensuring students have enhanced learning experiences during their ward rotations [19].

Based on the study’s findings, several recommendations emerge to enhance the learning environment in orthopaedic wards for undergraduate medical students. Firstly, there’s a need for faculty development programs focusing on student engagement strategies to address the perceived lack of faculty interest in students’ learning. This involves emphasizing personalized attention and engagement with students. Improving teaching quality is also crucial, necessitating targeted training for clinical teachers in effective teaching methods, hands-on clinical skills, and constructive feedback techniques.

Given the differing levels of satisfaction between male and female students, it’s vital to create a more gender-inclusive learning environment. This includes understanding and catering to the specific needs of all genders and ensuring equitable learning opportunities. Attention should also be given to the varying experiences of junior and senior students, suggesting the need for continuous curriculum assessment and adaptation to maintain high satisfaction levels as students progress in their studies.

The significance of positive staff attitudes and effective role modelling in enhancing student satisfaction cannot be overstated. Regular workshops and training sessions focusing on professional conduct and positive interactions with students in clinical settings are recommended. Moreover, implementing a system for regular student evaluations and feedback regarding their clinical rotation experiences will help in making continual improvements.

Aligning learning objectives with the tasks assigned to students during clinical rotations ensures that these tasks are appropriate and contribute meaningfully to their learning goals. Additionally, the unique challenges of learning in orthopaedic wards, such as balancing service provision and teaching, should be acknowledged and addressed, potentially by restructuring rotations or increasing teaching staff.

Finally, there’s a clear need for expanded research into clinical learning environments, particularly in less studied areas, to continuously refine and improve medical education. These comprehensive recommendations aim to significantly enhance the educational experience of medical students, fostering their academic and professional growth in clinical settings.

## Limitations

As a limitation of the study, it should be noted that the research relies solely on students’ subjective perceptions of the orthopaedic ward’s learning environment. It is a cross-sectional study conducted at a single centre, so its findings are not generalizable. Additionally, the study does not delve into the underlying causes of the students’ positive or negative views about the clinical learning environment.

## Conclusion

This study’s results indicated that the undergraduate students had an optimistic perception of the learning environment in the orthopaedic ward placement. Male participants had a more positive experience than their female counterparts. Moreover, staff attitudes are equally crucial to the quality of teaching and creating a productive learning environment in an educational setting. The medical institute must provide and maintain a safe and effective learning environment in wards that lay the foundation for necessary clinical competencies learning and promote the highest level of patient care. The findings of this study can be used to improve students’ learning experience in the orthopaedic ward and produce skilled doctors who will benefit the healthcare system overall.

## Supporting information

S1 File Dataset. (XLSX)
